# Ankylosing spondylitis and glaucoma in European population: A Mendelian randomization study

**DOI:** 10.3389/fimmu.2023.1120742

**Published:** 2023-03-20

**Authors:** Shengjie Li, Minting Chen, Qing Zhang, Meijin Fang, Wei Xiong, Lang Bai

**Affiliations:** ^1^ Department of Ophthalmology, Nanfang Hospital, Southern Medical University, Guangzhou, China; ^2^ Department of Thoracic Surgery and Oncology, The First Affiliated Hospital of Guangzhou Medical University, China State Key Laboratory of Respiratory Disease and National Clinical Research Center for Respiratory Disease, Guangzhou Institute of Respiratory Disease, Guangzhou, China

**Keywords:** ankylosing spondylitis, glaucoma, causal relationship, risk factor, Mendelian randomization

## Abstract

**Background:**

The relationship between ankylosing spondylitis (AS) and glaucoma in the European population remains unclear. In the present study, we applied a two-sample Mendelian randomization (MR) method to investigate their causal relationship.

**Methods:**

MR analysis was conducted to validate the causal associations between AS with glaucoma using summary statistics from the genome-wide association studies of AS (9,069 cases and 13,578 control subjects) and glaucoma (8,591 cases and 210,201 control subjects). The inverse variance weighting method was performed to evaluate the causal relationship. The MR–Egger regression approach was applied to assess pleiotropy, while Cochran’s Q test was used to analyze heterogeneity. Subgroup analysis was performed according to primary open-angle glaucoma (POAG) and primary angle-closure glaucoma (PACG).

**Results:**

The results of the MR study reveal a risk-increasing causal relationship between AS and glaucoma among European populations (OR = 1.35, 95%CI = 1.16–1.57, *P* = 8.81 × 10^-5^). Pleiotropy and heterogeneity were not found in our study. In the subgroup analysis, AS was also causal with POAG (OR = 1.48, 95%CI = 1.17–1.86, P = 8.80 × 10^-4^) and PACG (OR = 1.91, 95%CI = 1.03–3.51, P = 3.88 × 10^-2^).

**Conclusion:**

The results of the MR analysis suggested a causal relationship between AS and glaucoma in the European population. Further studies are needed to identify the specific mechanism between these two diseases.

## Introduction

Glaucoma is a neurodegenerative disease characterized by the atrophy and depression of the optic disc and the defects of visual field and vision (1). Tham et al. reported a global prevalence of glaucoma of 3.54% in adults aged 40–80 years, of which the prevalence of primary open-angle glaucoma (POAG) was 3.05%, and it is estimated that, by 2040, the number of glaucoma patients worldwide will increase to 111.8 million (2). Glaucoma is the leading cause of irreversible blindness, with disease burden varying based on glaucoma subtypes and ethnicity. Without prompt treatment, it can result in irreversible visual impairment (3, 4). Pathological elevated intraocular pressure is the main risk factor for glaucoma (5). However, it cannot explain all glaucoma-related symptoms. Even after intraocular pressure (IOP) recovers to normal, some patients continue to acquire progressive visual field defects and optic atrophy. Therefore, early detection and diagnosis are undoubted of great significance to glaucoma.

Autoimmunity, inflammation, oxidative stress, mitochondrial dysfunction, and retinal ischemia have all been reported to be the risk factors for glaucoma (6–10). Several pieces of research have implicated antibodies and CD4^+^ T cells and the pathogenesis of glaucoma (11, 12). An increasing number of studies have unmasked the link between autoimmune diseases and glaucoma.

As an autoimmune disease, ankylosing spondylitis (AS) is a chronic arthritis characterized by the inflammation of the spine and sacroiliac joints (13, 14). AS affects the eyes through immune, cytokine, and infection mechanisms. There are 15.8% of AS patients who have the ocular disease (15). Uveitis is the most prevalent extra-articular manifestation, and there exists a correlation between certain ocular diseases and AS. Patients with optic neuritis are a high-risk population for autoimmune diseases, including AS (16, 17). However, due to limited data on AS cases, there are few studies on the association between AS and glaucoma (even primary glaucoma). Most studies on the relationship between AS and glaucoma focus only on the fact that AS causes uveitis, which further leads to secondary glaucoma. Clinicians often consider the possibility of AS in the face of uveitis patients in clinical practice. Lea et al. found that Europeans with the interleukin (IL)-38 polymorphism rs3811058 (IL-1F10.3) were more likely to develop AS (18). Recent studies have reported that IL-38 is involved in inflammatory autoimmune diseases, including AS and glaucoma (19). Furthermore, IL-36, IL-37, and IL-38 may be involved in the immune-mediated pathogenesis of chronic primary angle-closure glaucoma (PACG) and are associated with a mean deviation of visual fields (19, 20). Existing evidence suggests an association between them. Nonetheless, no studies have reported a direct causal relationship between AS and glaucoma.

Mendelian randomization (MR) analysis, which incorporates genetic variation as an instrumental variable (IV), has been frequently used to evaluate the potential causal association between risk factors and disease results (21, 22). Accordingly, MR analysis views genetic variation as a natural experiment entailing the random assignment of alleles associated with exposure during the time of gamete formation, in accordance with Mendel’s second law, which is typically independent of environmental risk factors and precedes risk factors and disease progression (23). More specifically, MR analysis avoids bias due to confounding or reverse causality as the genotype cannot be altered by the disease (24). Accordingly, MR analysis is less susceptible than traditional observational studies to confounding, reverse causality, and measurement error (25). By taking single-nucleotide polymorphisms (SNPs) as genetic variants, this approach bears a tremendous advantage in that the SNP-exposure effects and SNP-outcome effects are obtained from separate studies. With these summary data alone, even if they are not measured in the same set of samples, it is possible to estimate the causal influence of the exposure on the outcome (26). In the absence of observational data on both, we were still able to apply the two-sample MR analysis for the purpose of exploring the causal relationship between AS and glaucoma. Consequently, the aim of our study was to apply a two-sample MR analysis in order to identify whether AS is causally associated with the risk of glaucoma.

## Methods

### Study design

The primary research of the genome-wide association studies (GWASs) from each nation had previously gained ethical consent, and the present analysis was based on publicly available summary-level data, which did not need additional approval. The publicly available summary data from the MR-Base platform were used in this study to assess the relationship between AS and glaucoma (26). To reduce the influence of population stratification, we restricted our study population to the individuals of European ancestry.

### Selection of genetic instruments for ankylosing spondylitis

For the present study, we employed SNPs associated with AS at the genome-wide significance threshold from *Cortes et al.’s* GWASs (27). This GWAS comprised 9,069 cases and 13,578 controls from 22,647 subjects of European ancestry. The association between each AS-contained SNP [p< 5 × 10^−8^; linkage disequilibrium (LD) r^2^< 0.001] was evaluated, and no LD was found. If a requested SNP was absent, the proxy SNP (LD at r^2^>0.8) from the 1000 Genomes Project was employed as the surrogate effect allele. In this case, LD proxy lookups are automatically provided by MR-Base (26). In addition, we removed the SNPs with the presence of palindromes. Eventually, 24 SNPs as genetic variables were conducted into the final IV set (**Supplementary Table S1**, **Supplementary Figures S1**, **S4**, **S7**).

### Data of genetic instruments for glaucoma

**Figure 1:**
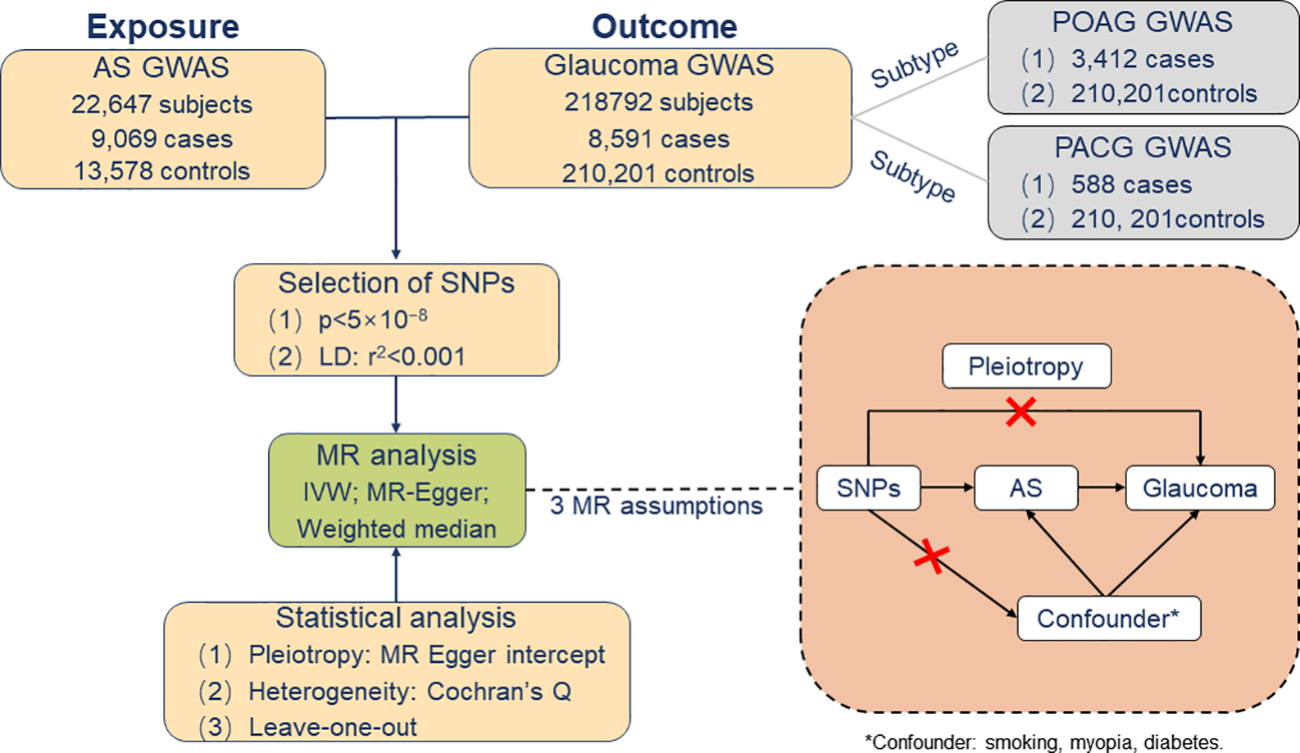
Overview of Mendelian randomization analysis and major assumptions. AS, ankylosing spondylitis; GWASs, genome-wide association studies; POAG, primary open-angle glaucoma; PACG, primary angle-closure glaucoma; *Confounder: smoking, myopia, diabetes.

In order to avoid the inclusion of data from the same participant, the Data of Genetic Instruments for glaucoma was selected from the FinnGen consortium, with a total of 218,792 individuals of European ancestry (including 8,591 glaucoma cases and 210,201 controls) (28). Furthermore, POAG (3,412 cases and 210,201 controls) and PACG (588 cases and 210,201 controls) were included as histological subgroups for further comprehension of the causality between genetically predisposed AS and glaucoma subtypes. This study defines glaucoma by H40-H42 of the International Classification of Disease-10, and other studies have supported the use and accuracy of the International Classification of Disease coding for this diagnosis (28, 29) (**Figure 1**).

Assessment of the Mendelian randomization assumptions

The following three assumptions must be fulfilled for the two-sample MR analysis to produce a correct interpretation: (I) SNPs are strongly associated with AS, usually, to satisfy this condition, with a p-value less than 5 × 10^−8^; (II) the IVs do not influence glaucoma through other pathways than the AS; and (III) the instrument variables do not associate with confounders. In addition, there is some potential pleiotropy in instrumental effects, which may indicate potential violations of the assumptions underlying two-sample MR. Therefore, other sensitivity analyses were carried out using multiple imputation methods to test the robustness of MR results. Accordingly, the first assumption can be quantified by the strength of an association between a genetic variant and the risk factor. On top of that, we calculated the F-statistic to measure the strength of IVs (30). This is related to the proportion of variance in the phenotype explained by the genetic variants (R^2^). The phenotype variance explained (R^2^) was obtained from GWAS databases or original literature. If the R^2^ were not provided in the GWAS databases or literature, we would assess the strength of instruments based on ${\text{β}}_{\text{exposure}}^{2}$ or ${\text{SE}}_{\text{exposure}}^{2}$ . Subsequently, to verify the second assumption, we performed directional pleiotropy based on the intercept obtained from the MR–Egger regression analysis (31, 32). Concerning the third hypothesis, once these selected R^2^ were associated with confounding factors for AS and glaucoma, our MR analysis would prove unable to estimate the causal effect of AS on glaucoma accurately. Consequently, we manually searched in all the published GWASs whether AS-related SNPs have secondary phenotypes other than AS. The cut-off date was on 30th August 2022. In addition, smoking represents a known risk factor for greater disease activity and worse functioning in AS patients (33). Simultaneously, a number of studies have reported that smoking intensity is associated with the faster thinning of the retinal nerve fiber layer (34). In consequence, smoking may be a potential confounding factor in the relationship between AS and glaucoma (35). In addition, epidemiologic studies show that both myopia and diabetes may be risk factors for glaucoma (36–38). It cannot be ruled out that confounding factors such as myopia and diabetes may further affect glaucoma by influencing AS. Hence, we selected these three assumptions as possible confounding factors to study AS (**Figure 1**).

### Statistical analysis

Inverse variance weighting (IVW) is generally regarded as the main method in MR analysis to test the causality between exposure and the outcome: in the absence of pleiotropy (and assuming that the instruments are valid), IVW returns the unbiased estimates of a causal effect so long as horizontal pleiotropy is balanced (26, 31). Accordingly, if there was just one SNP, we estimated the causal effect of AS on glaucoma using the Wald ratio method (22). In addition, we estimated the effects using the weighted median (WM) and MR–Egger methods (26). In this regard, the MR–Egger method has the advantage of allowing horizontal pleiotropic effects. Accordingly, if the results are consistent with the IVW method, then, confidence is gained in the conclusions. In this study, power calculations were performed based on a method suggested by Burgess et al. to confirm whether the IVs provided relatively accurate estimates of causal effects (39).

Finally, we performed Cochran’s Q test for the assessment of heterogeneity in order to assess whether the estimate of the causal effect of AS on glaucoma was consistent across each SNP (40). On top of that, a leave-one-out analysis was conducted to estimate whether the result was biased or driven by a single SNP, which sequentially excluded one SNP at a time, in order to assess the sensitivity of the results for individual variants (41).

MR analysis was performed in R (version 3.6.3) with the package “TwoSampleMR” (version 0.5.6).

## Results

### Mendelian randomization analysis of ankylosing spondylitis with risk of glaucoma and subtypes

Our results showed that AS was causally correlated with a markedly increased glaucoma risk among European populations. IVW was used to indicate that AS (OR = 1.35, 95%CI = 1.16–1.57, P = 8.81 × 10^-5^) was associated with the risk of glaucoma. We further validated the results of IVW by WM (OR = 1.42, 95%CI = 1.15–1.74, P = 1.08 × 10^-3^) and MR–Egger (OR = 1.33, 95%CI = 1.03–1.73, P = 3.97 × 10^-2^), and the results are similar. In addition, in the subgroup analysis, AS was found to be causally associated to POAG (OR = 1.48, 95%CI = 1.17–1.86, P = 8.80 × 10^-4^) and PACG (OR = 1.91, 95%CI = 1.03–3.51, P = 3.88 × 10^-2^), respectively, and these associations were consistent in the methods of WM and MR–Egger, with similar causal estimates in direction and amplitude (**Table 1**, **Supplementary Figures S2**, **S5**, **S8**).

**Table 1:** Mendelian randomization (MR) results between ankylosing spondylitis (AS) and glaucoma.

Outcome	IVW		MR–Egger		Weighted median	
	OR (95% CI)	P-value	OR (95% CI)	P-value	OR (95% CI)	P-value
Glaucoma	1.35 (1.16, 1.57)	8.81E-05	1.33 (1.03, 1.73)	3.97E-02	1.42 (1.15, 1.74)	1.08E-03
POAG	1.48 (1.17, 1.86)	8.80E-04	1.74 (1.17, 2.59)	1.21E-02	1.55 (1.12, 2.15)	8.27E-03
PACG	1.91 (1.03, 3.51)	3.88E-02	1.43 (0.49, 4.15)	0.520	1.41 (0.65, 3.04)	0.380

MR, Mendelian randomization; AS, ankylosing spondylitis; POAG, primary open-angle glaucoma; PACG, primary angle-closure glaucoma.

### Verification of the Mendelian randomization assumptions

#### F-statistic

We assessed the strength of instruments by an F-statistic. The F-statistic of AS was much larger than 10, indicating that the instrument accurately predicted glaucoma.

#### MR–Egger pleiotropy test

Furthermore, the impact of pleiotropy of the exposures given the intercept value could be negligible as no evidence for the presence of directional pleiotropy in the MR–Egger regression analysis was found (interrupt β<0.001, P = 0.92) (**Table 2**).

**Table 2:** MR–Egger pleiotropy test.

Outcome	MR–Egger method	
	Intercept	P-value
Glaucoma	0.00067	0.92
POAG	-0.01023	0.33
PACG	0.01804	0.52

POAG, primary open-angle glaucoma; PACG, primary angle-closure glaucoma.

### Causal effect from ankylosing spondylitis on potential glaucoma risk factors

For the third hypothesis, we found no evidence that the included AS-associated SNPs have secondary phenotypes other than AS during our manual search. Moreover, the overall correlations between AS and confounders (smoking, myopia, and diabetes) were not statistically significant (**Table 3**).

**Table 3:** MR results between AS and confounding factors.

Outcome	SNPs	IVW	
		OR (95% CI)	P-value
Cigarettes smoked per day	22	1.00 (0.47, 2.11)	0.998
Myopia	24	1.20 (0.87, 1.65)	0.262
Type 2 diabetes	25	1.07 (0.85, 1.34)	0.567

MR, Mendelian randomization; AS, ankylosing spondylitis; SNPs, single-nucleotide polymorphisms; IVW, inverse variance weighted.

### Power calculation

Under the supposition of the 24 selected SNPs explaining a total of 24.4% variance of AS, the MR analysis had 100% power at a significance level of 0.05 to detect the previously estimated causal effect size of AS (OR = 1.35), suggesting a more reliable estimation of the causal effect.

### Heterogeneity and leave-one-out sensitivity analysis

According to the results of Cochran’s Q test, there was no heterogeneity among these SNPs (**Supplementary Table S2**). Leave-one-out sensitivity analysis could be found in the appendix. It seemed that no single SNP was strongly driving the overall effect of each exposure on glaucoma (**Supplementary Figures S3**, **S6**, **S9**).

## Discussion

In our study, MR analysis revealed a significant causal relationship between AS and glaucoma, which was the first large-scale MR study to investigate the correlation between AS and glaucoma. This study provided a better understanding of potential risk factors for glaucoma in AS patients.

Ocular involvement is one of the most common extra-articular manifestations in AS patients. The pathogenesis of ocular disease in AS patients has yet to be elucidated. The fact that 15.8% of AS patients will develop anterior uveitis makes ophthalmologists pay more attention to AS in clinical practice, which is related to human leukocyte antigen (HLA)-B27 and the disease duration of AS (42). AS has an increased risk of developing chronic diseases such as diabetes mellitus and cardiovascular and cerebrovascular diseases (43). However, AS patients with acute anterior uveitis (AAU) did not increase the risk of subsequent major adverse cardiovascular events compared to non-AAU controls (44). The mechanism of how AS modifies glaucoma risk remains unclear. According to certain studies on the association between AS and glaucoma, corneal hysteresis (CH) reduced as the duration of AS increased. CH is considered a risk factor for all types of glaucoma and progressive glaucoma optic neuropathy because it represents the ability of the corneal stromal tissue to absorb and disseminate energy (45, 46). Furthermore, central corneal thickness (CCT) values reduced with increasing Bath Ankylosing Spondylitis Metrology Index score, which may result in lower IOP power during measurement, while thinner CCT with ocular hypertension is more prone to glaucoma (5, 47).

It is worth noting that the MR analysis conducted in our study basically satisfied three key assumptions (validated by sensitivity analysis), which can remove the influence of most confounding factors and avoid reverse causality. MR analysis has given us a better understanding of potential risk factors for glaucoma.

In the current clinical work, the conventional treatment of glaucoma has been directed toward controlling IOP, primarily because that is the proven modifiable risk factor for glaucoma. Topical drug therapy, which has been commonly used in the past, is being challenged by selective laser trabeculectomy, microinvasive glaucoma surgery, and sustained delivery methods. Surgery and drugs to reduce intraocular pressure and protect optic ganglion cells are effective ways to save the vision of glaucoma patients (48). However, early detection and intervention of glaucoma are still essential. The data analysis of medical therapy for the normotensive fellow eye of dogs previously diagnosed with primary glaucoma suggests that utilizing a carbonic anhydrase inhibitor and a prostaglandin analog would be reasonable (49). Individualized, refined preventive measures are beneficial for patients with high-risk factors for glaucoma. In other words, it is critical for patients with AS to assess their physical status and manage modifiable risk factors in glaucoma from a public health perspective. With the increasing availability of a wealth of genetic data, the expansion of GWASs could lead to the realization of early detection of glaucoma and aid in the diagnosis of AS, potentially enabling genetic-based treatments (4, 50).

The association between AS and glaucoma is influenced by a number of intermediary phenotypes, which must be taken into account. It is widely known that anterior uveitis is an important clinical feature of AS, and its complications may lead to secondary glaucoma. The MR analysis revealed a clear causal association between AS and uveitis, as well as an inseparable correlation between uveitis and secondary glaucoma, which is an important intermediary factor when analyzing the relationship between uveitis and secondary glaucoma. However, the AS–uveitis–secondary glaucoma axis may be a significant confounding effect in MR analysis on the relationship between AS and primary glaucoma. Based on the nature of the data we used, AS could not be stratified by uveitis. In this study, we were also unable to separate AS from confounding factors. Therefore, we performed an MR analysis between AS and primary glaucoma to strengthen the validation of the causal relationship between AS and glaucoma. The results suggested that uveitis could not fully explain the relationships between AS and glaucoma, while internal mechanisms have not been clearly unmasked. In the analysis of the relationship between AS and glaucoma, uveitis complicated by AS has provided a mechanistic explanation that does impact the numerical results, even though this is within the expected range.

Furthermore, we should keep in mind that there are still potential confounding factors in the study of AS and glaucoma, such as age, gender, medication use, and the duration of the type of glaucoma exposure. However, further analysis was difficult to conduct as the corresponding GWAS data were not available. In theory, we cannot rule out all possible confounds that may occur in MR analysis. The MR analysis (closest to a randomized controlled experiment) remains the most effective method for determining causality in the presence of confounders in the face of fewer research data and a lower likelihood of conducting a randomized controlled trial as it can minimize the effects of confounders and provides sufficient statistical power for causal estimation.

There are also limitations to MR analysis. Firstly, our MR analysis included only 24 SNPs in published GWAS. Secondly, the samples involved in our study were limited to European populations, which may not apply to Asian or African populations who have a higher incidence of glaucoma. Therefore, larger sample studies with more AS-related SNPs and more ethnic groups may help provide more valid conclusions about the causal relationship between AS and glaucoma. Furthermore, genetic variation in the analysis can explain only a small amount of individual diversity. In other words, in social epidemiological investigations, the mean of the relevant group must be evaluated rather than the mean of the individual. Finally, we were unable to stratify the population according to medication usage, disease duration, the history of uveitis, and other factors to avoid potential biases caused by the aforementioned confounding factors, and we cannot completely rule out the possible influence of pleiotropic effects on the results.

In conclusion, we conducted the first MR study and subgroup analysis to investigate the causal relationship between AS and glaucoma in the European population. Our study revealed a significant causal relationship between AS and glaucoma, especially POAG or PACG. We used MR analysis to mimic randomized controlled trials, which can avoid reversing causality and potential confounders common in conventional observational studies. However, the specific mechanism by which AS causes primary glaucoma requires further investigation.

## Data availability statement

The original contributions presented in the study are included in the article/**Supplementary Material**. Further inquiries can be directed to the corresponding author.

## Author contributions

SL: Conceptualization, Data Curation, Methodology, Formal analysis, Writing- Original draft, Writing-Reviewing and Editing. MC: Data Curation, Methodology, Formal analysis, Writing- Original draft. QZ: Formal analysis, Writing-Reviewing and Editing. MF: Methodology, Formal analysis. WX: Methodology, Formal analysis. LB: Conceptualization, Supervision, Validation, Writing-Reviewing and Editing. All authors contributed to the article and approved the submitted version.
