# Supplementary material for: Ankylosing spondylitis and glaucoma in European population: A Mendelian randomization study
Source: Front Immunol. 2023 Mar 20;14:1120742. doi: 10.3389/fimmu.2023.1120742 (PMC10067563; doi:10.3389/fimmu.2023.1120742)
Supplement: Supplementary file 3 [file DataSheet_1.pdf]

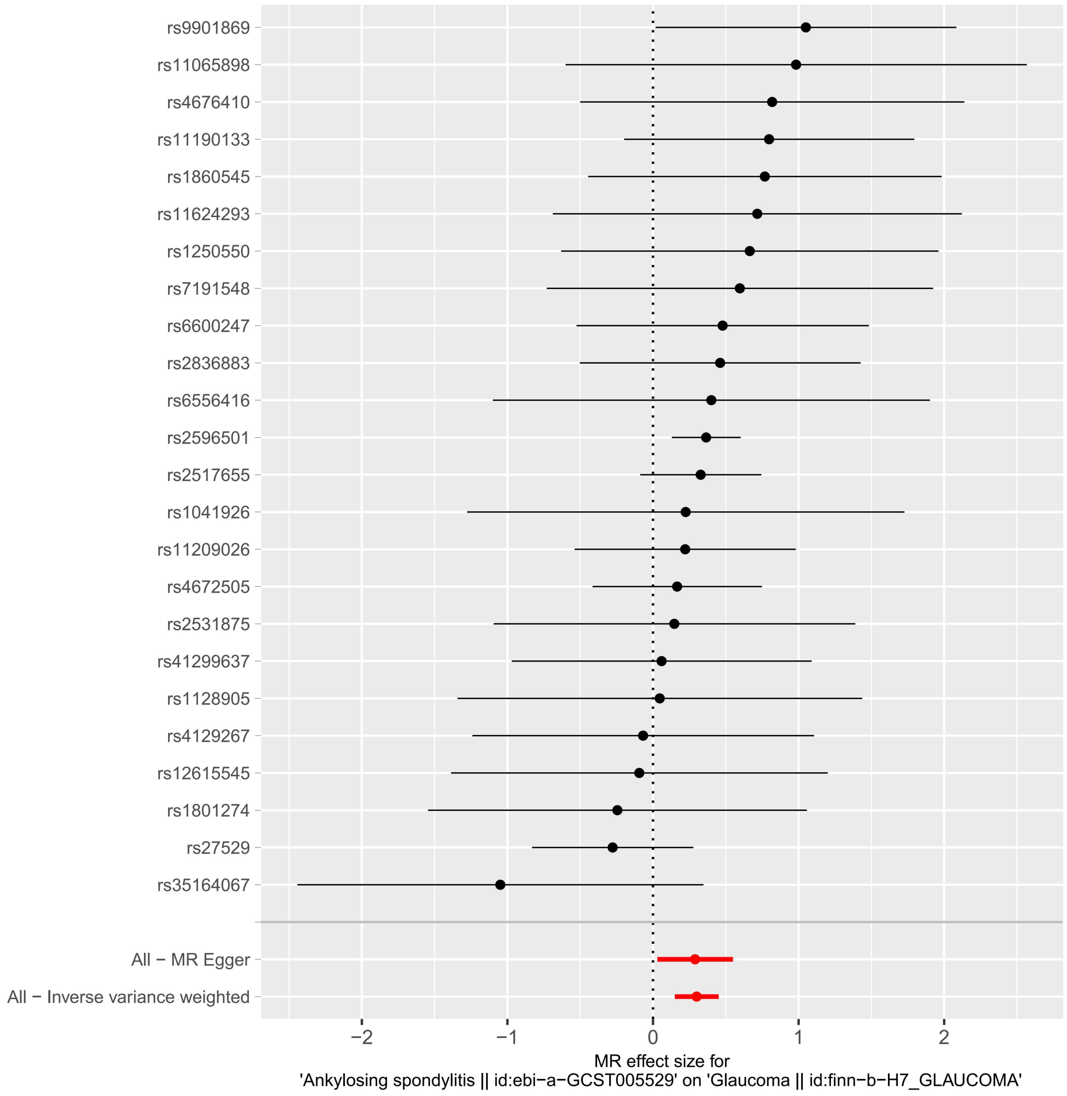

Figure S1. Forest plot of SNPs associated with AS and their risk of glaucoma.

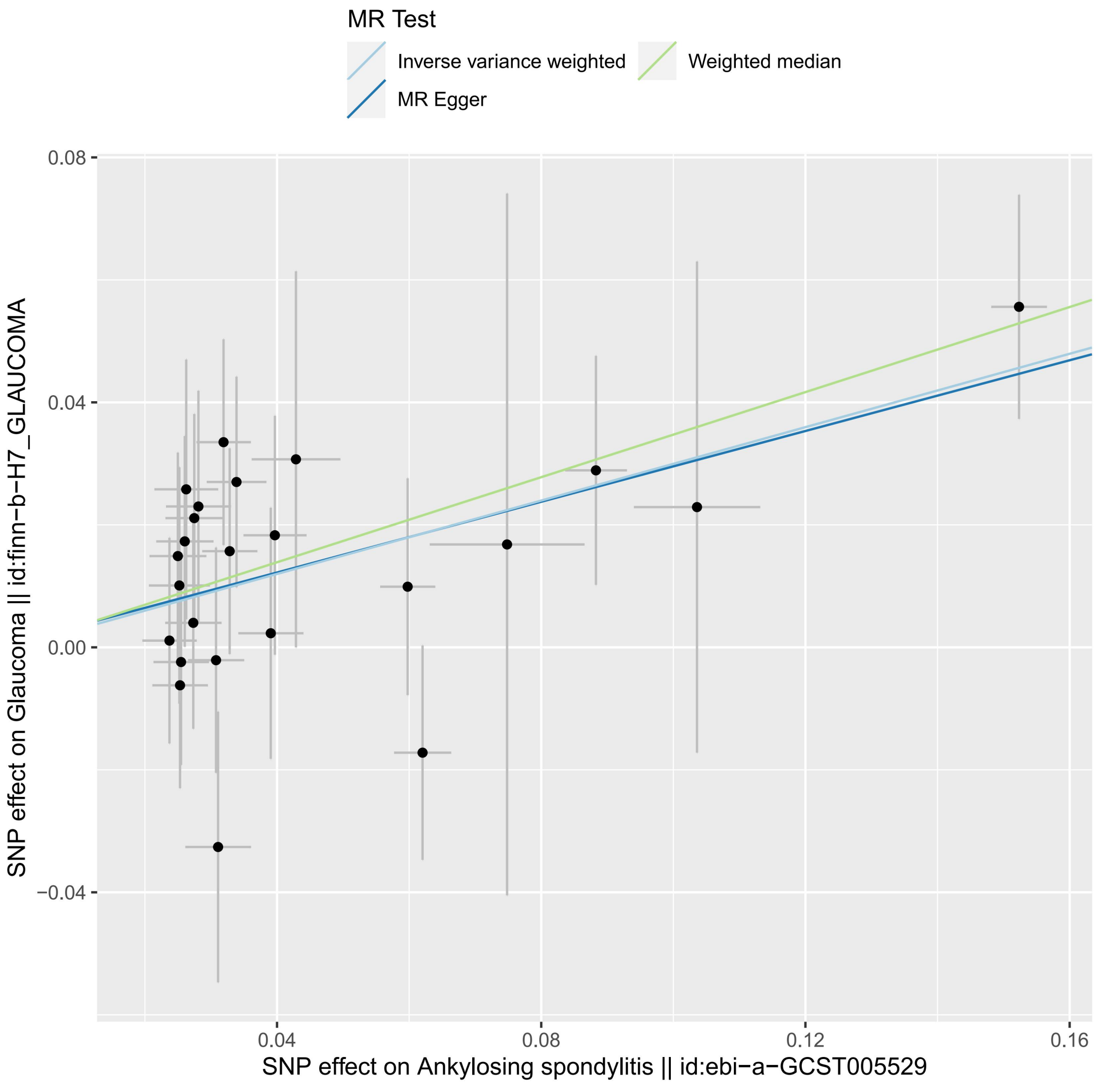

Figure S2. Scatter plot of SNPs associated with AS and their risk of glaucoma.

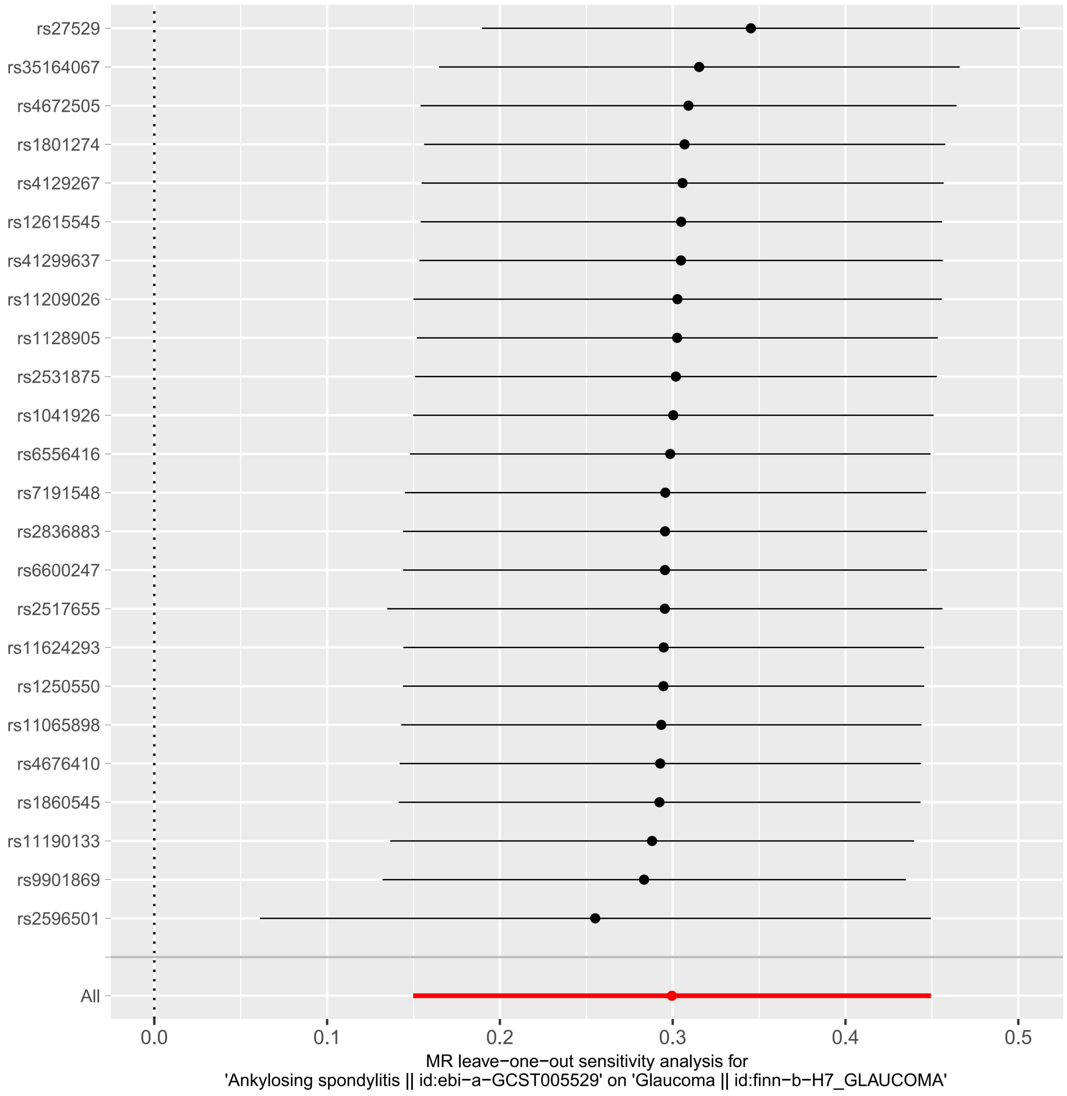

Figure S3. Leave-one-out of SNPs associated with AS and their risk of glaucoma.

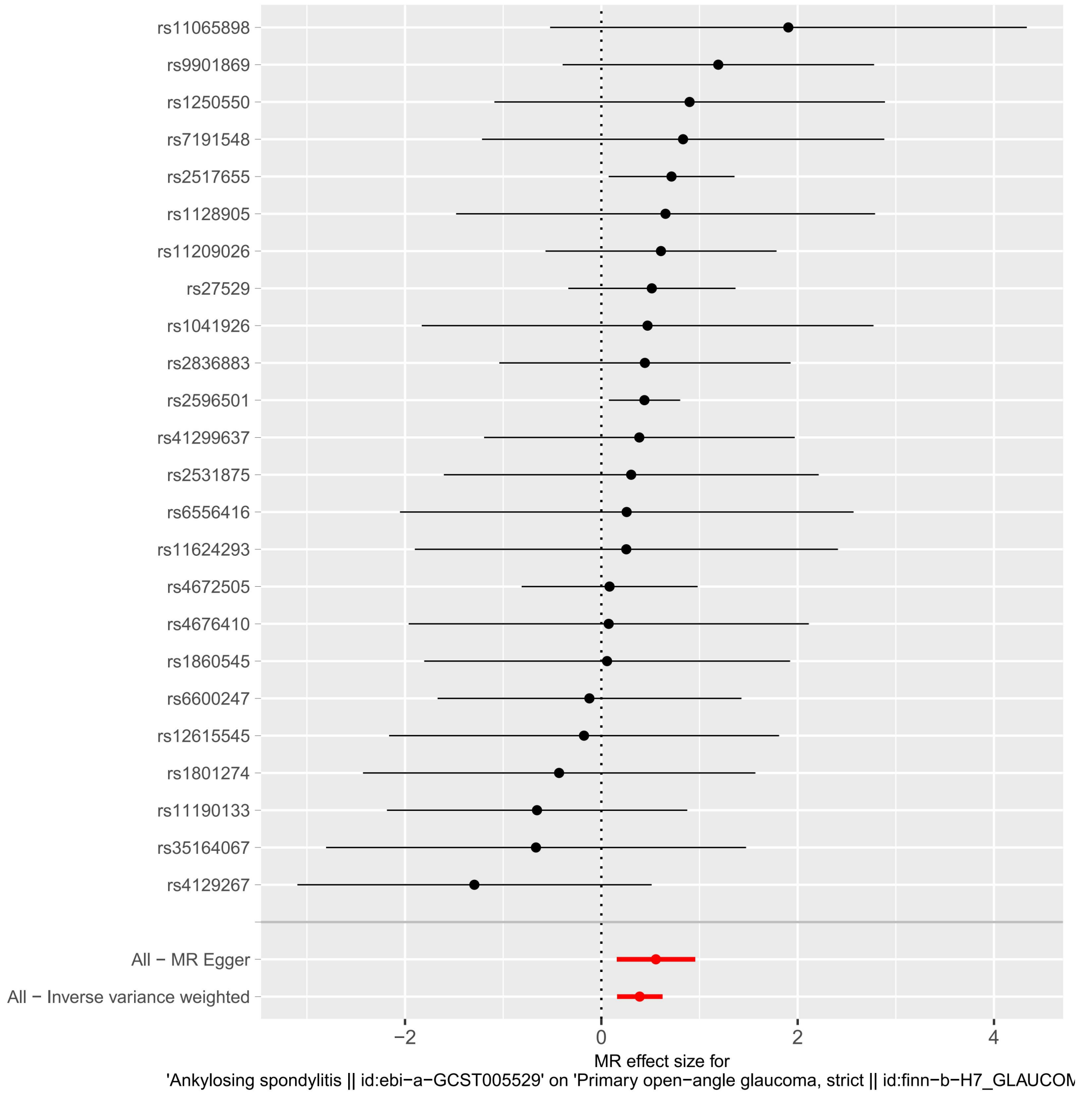

Figure S4. Forest plot of SNPs associated with AS and their risk of POAG.

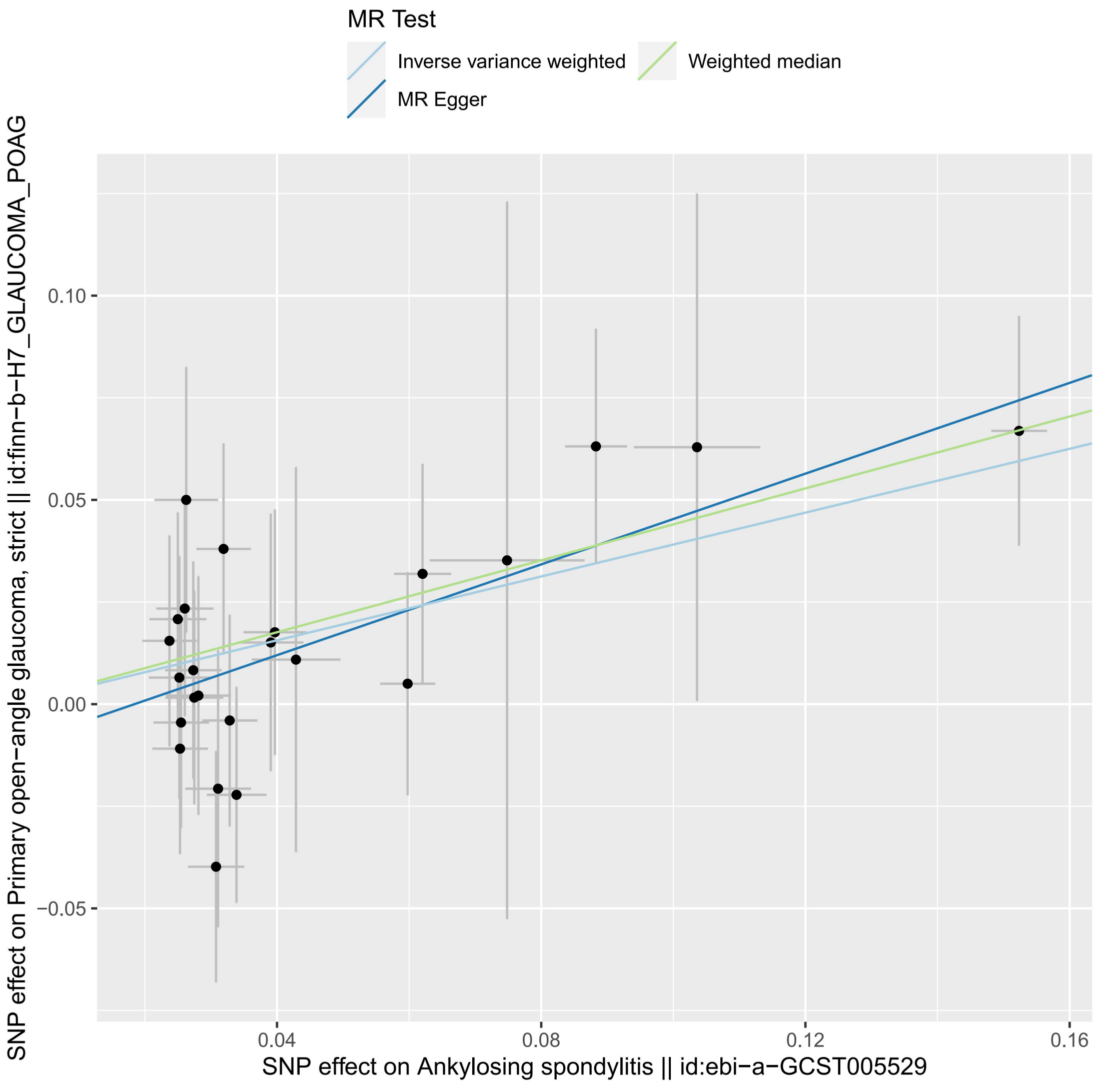

Figure S5. Scatter plot of SNPs associated with AS and their risk of POAG.

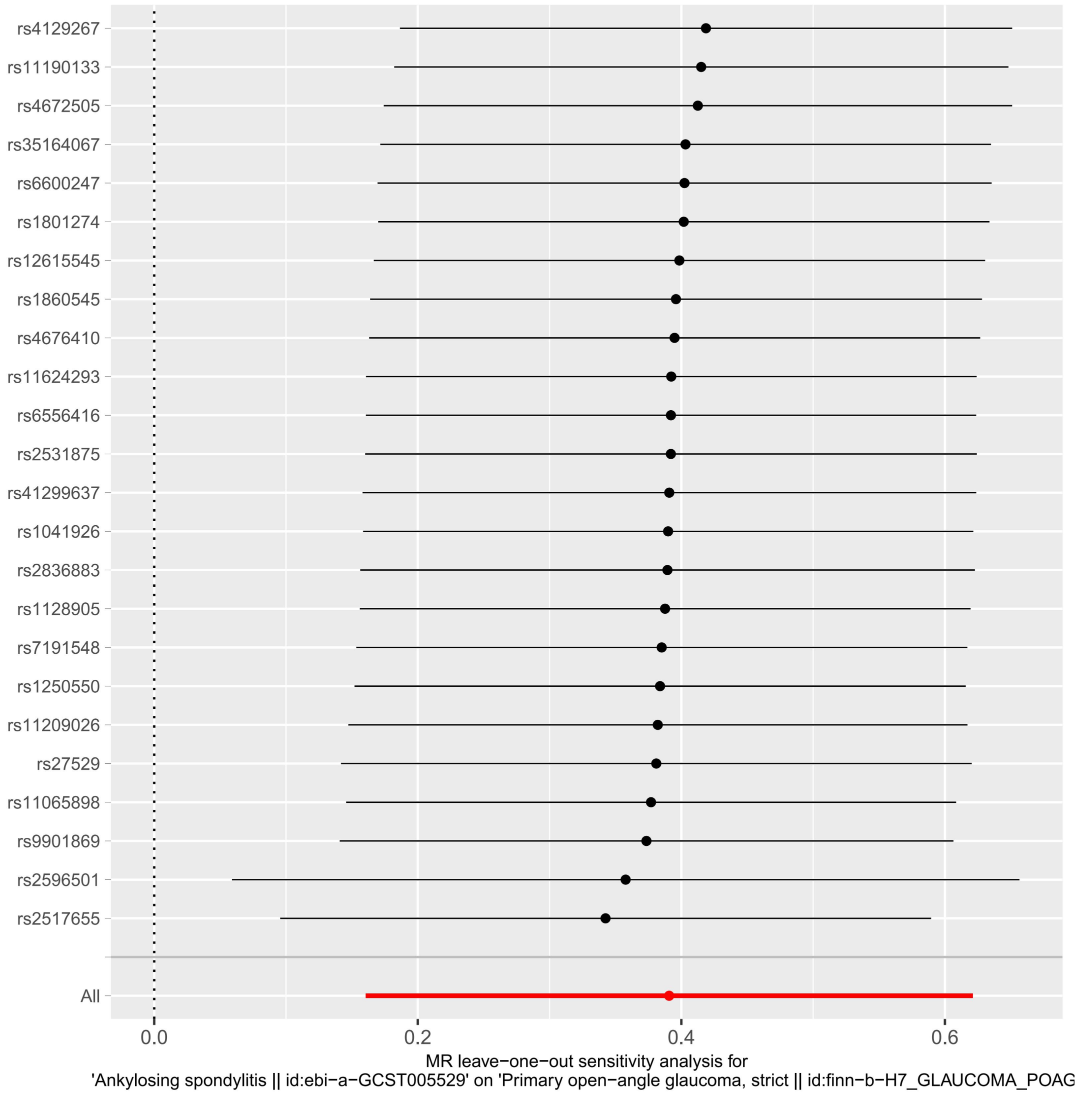

Figure S6. Leave-one-out of SNPs associated with AS and their risk of POAG.

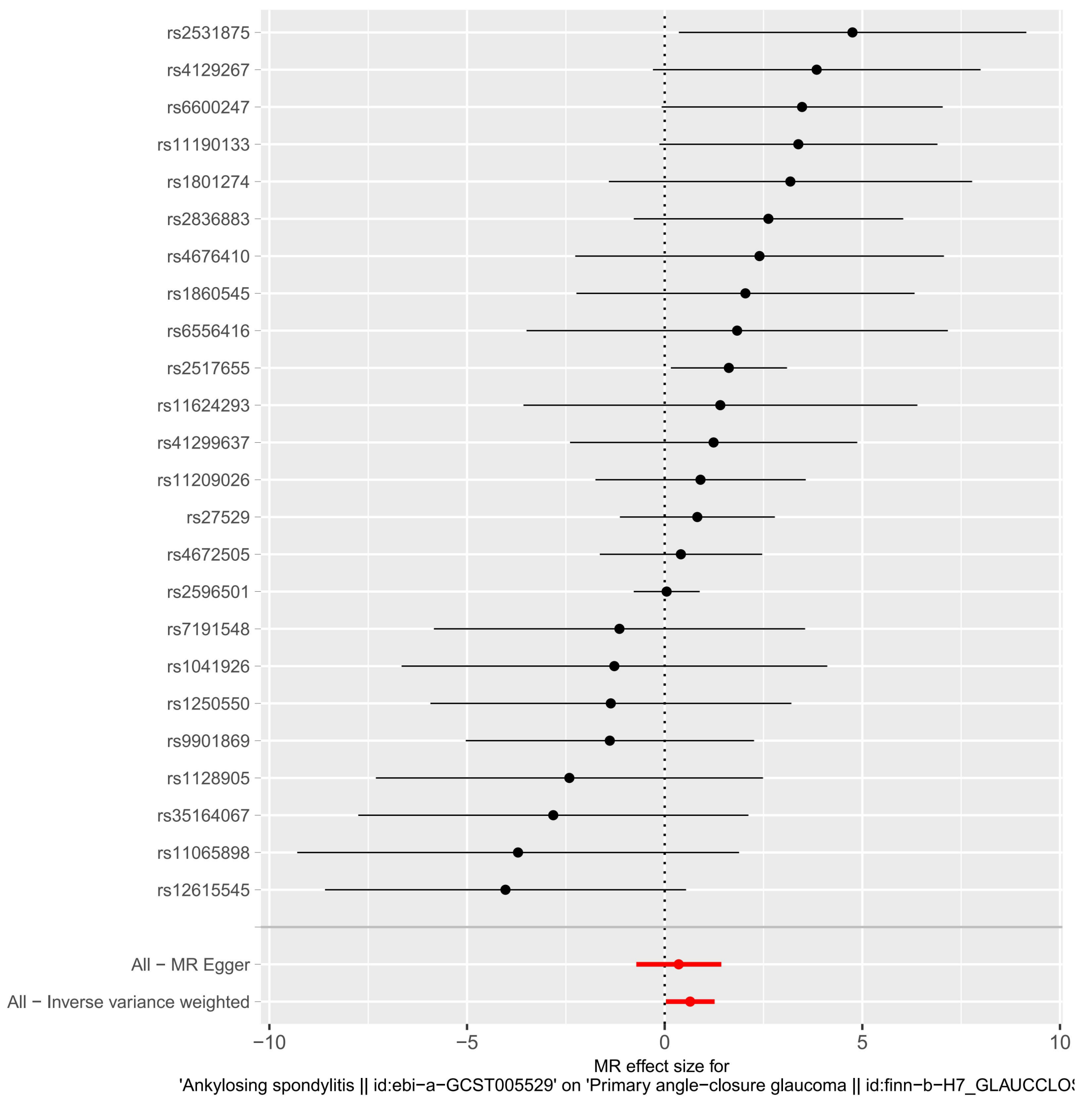

Figure S7. Forest plot of SNPs associated with AS and their risk of PACG.

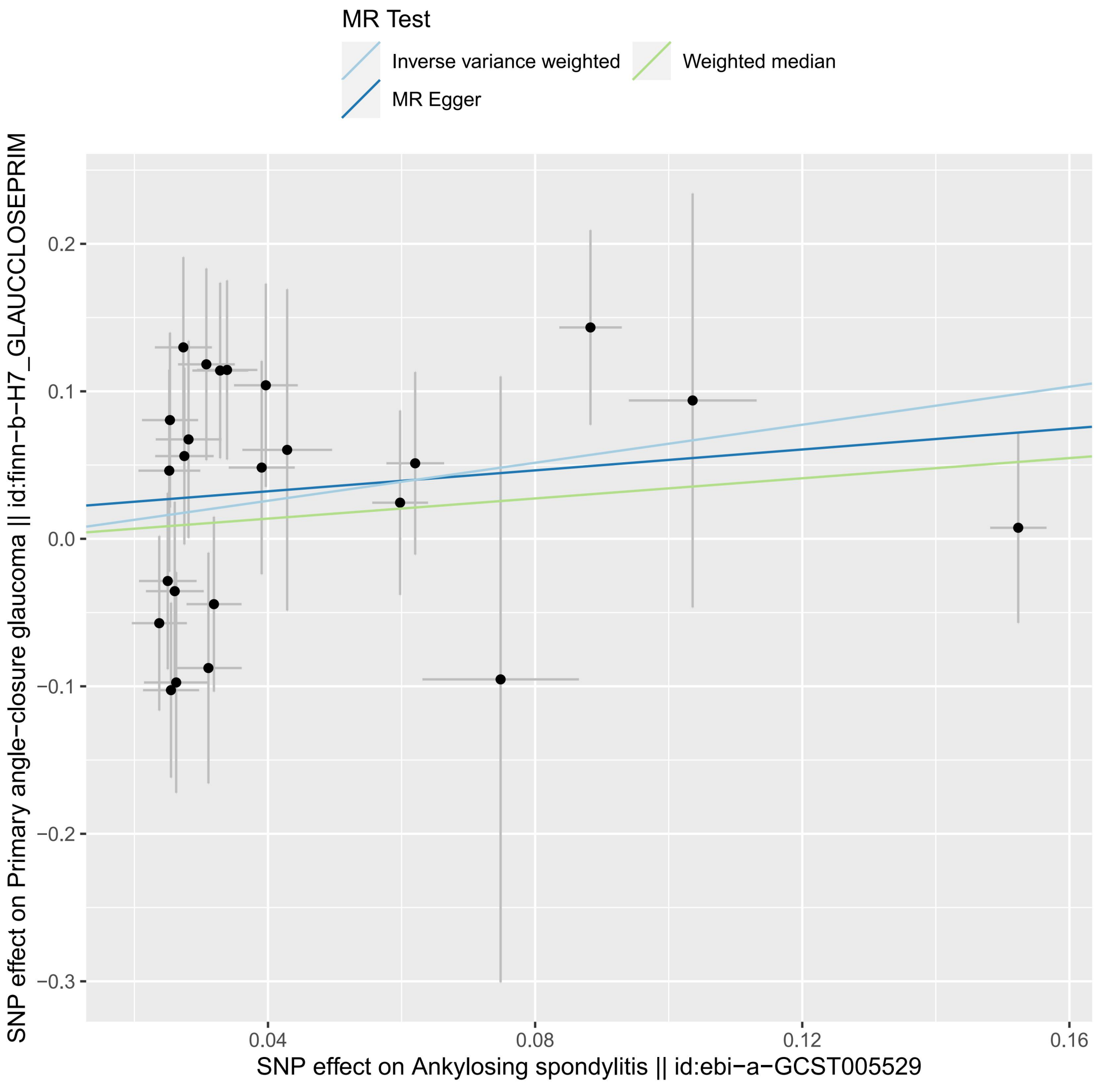

Figure S8. Scatter plot of SNPs associated with AS and their risk of PACG.

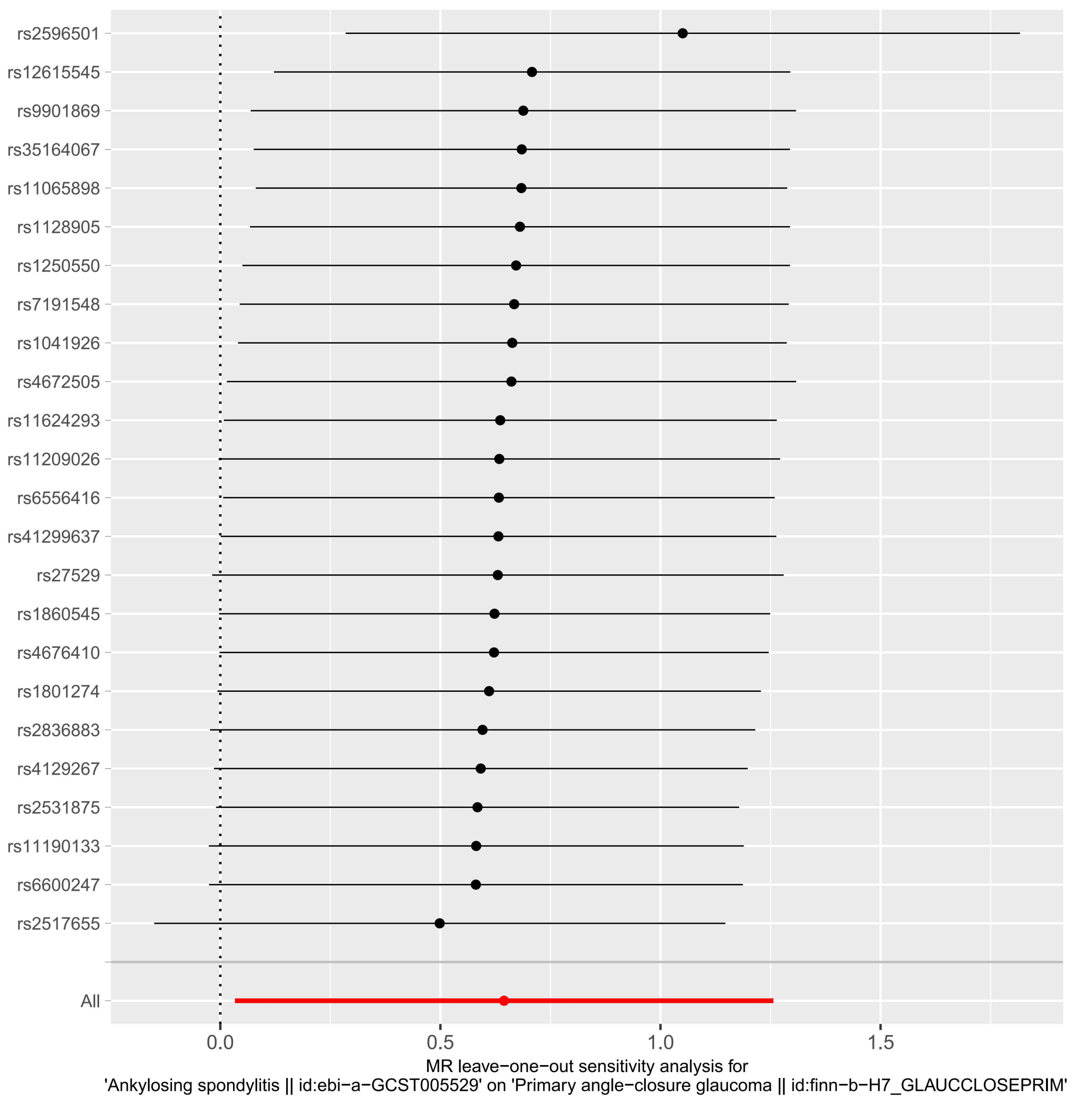

Figure S9. Leave-one-out of SNPs associated with AS and their risk of PACG.
